# Neuroepigenetic Mechanisms of Action of Ultrashort Peptides in Alzheimer’s Disease

**DOI:** 10.3390/ijms23084259

**Published:** 2022-04-12

**Authors:** Anastasiia Ilina, Vladimir Khavinson, Natalia Linkova, Mikhael Petukhov

**Affiliations:** 1 Department of Biogerontology, Saint Petersburg Institute of Bioregulation and Gerontology, 19711 Saint Petersburg, Russia; vladimir@khavinson.ru (V.K.); miayy@yandex.ru (N.L.); 2 Department of General Pathology and Pathological Physiology, Institute of Experimental Medicine, 197376 Saint Petersburg, Russia; 3 Group of Peptide Regulation of Aging, Pavlov Institute of Physiology, Russian Academy of Sciences, 199034 Saint Petersburg, Russia; 4 Department of Molecular Radiation Biophysics, Petersburg Nuclear Physics Institute Named after B.P. Konstantinov, NRC “Kurchatov Institute”, 188300 Gatchina, Russia; michael.petukhov@yandex.ru; 5 Group of Biophysics, Higher Engineering and Technical School, Peter the Great St. Petersburg Polytechnic University, 195251 Saint Petersburg, Russia

**Keywords:** ultrashort peptides, DNA, promotors, histones, nucleosome, DNA-binding proteins, non-coding RNA, transcription factors, neuroepigenetic, Alzheimer’s disease

## Abstract

Epigenetic regulation of gene expression is necessary for maintaining higher-order cognitive functions (learning and memory). The current understanding of the role of epigenetics in the mechanism of Alzheimer’s disease (AD) is focused on DNA methylation, chromatin remodeling, histone modifications, and regulation of non-coding RNAs. The pathogenetic links of this disease are the misfolding and aggregation of tau protein and amyloid peptides, mitochondrial dysfunction, oxidative stress, impaired energy metabolism, destruction of the blood–brain barrier, and neuroinflammation, all of which lead to impaired synaptic plasticity and memory loss. Ultrashort peptides are promising neuroprotective compounds with a broad spectrum of activity and without reported side effects. The main aim of this review is to analyze the possible epigenetic mechanisms of the neuroprotective action of ultrashort peptides in AD. The review highlights the role of short peptides in the AD pathophysiology. We formulate the hypothesis that peptide regulation of gene expression can be mediated by the interaction of short peptides with histone proteins, cis- and transregulatory DNA elements and effector molecules (DNA/RNA-binding proteins and non-coding RNA). The development of therapeutic agents based on ultrashort peptides may offer a promising addition to the multifunctional treatment of AD.

## 1. Introduction

Alzheimer’s disease (AD) is a neurodegenerative disease and the most common cause of dementia [1]. Memory loss and psychophysiological and motor disorders are observed in AD. These clinical signs are caused by multiple atrophy of the cerebral cortex, as well as the amygdala and hippocampus. The wide range of molecular and cellular AD-associated processes, such as neuroinflammation, oxidative stress, lipid and carbohydrate metabolism disorders, mitochondrial dysfunction, calcium imbalance, and impaired neurogenesis lead to the development of the above clinical symptoms [2].

In the scientific community, more and more convincing arguments are being made in favor of the important role of epigenetics in AD pathogenesis [3,4]. According to the latest data, such epigenetic mechanisms as chromatin remodeling, DNA methylation, and non-coding RNA (ncRNA) play an important role in AD pathophysiology [5]. This approach will expand the understanding of AD pathogenesis. It will identify new strategies for the therapeutic correction of this disease.

Modern drugs approved by the Food and Drug Administration (FDA) for the treatment of AD (memantine, tacrine, rivastigmine, galantamine, and donepezil) do not significantly slow down the progression of the disease [6]. In addition, these drugs are known to have side effects [7]. Therefore, the therapeutic strategy of targeted action on a single molecular target is probably insufficient to combat the multifactorial pathogenesis of AD [8].

There is evidence of a polypharmacological approach, which includes the use of pharmaceutical agents acting on several targets, which seems to be more suitable for the correction of AD [9]. Therefore, the search for therapeutic agents with a multifunctional mechanism of action is a promising strategy for the development of an effective drug for the treatment of AD. In this regard, the study of AD pathogenesis at the molecular genetic level will make it possible to identify multiple targets for the polypharmacological correction of the disease.

Peptides are promising neuroprotectors with a wide range of biological effects. Of particular interest is a group of ultrashort peptides consisting of 2–7 amino acid residues [10,11,12].

Peptides support many key processes in the body due to their antioxidant, antimicrobial, antibacterial, anti-inflammatory, anticarcinogenic, antitumor, and immuno-regulatory characteristics. We described biological properties of short peptides in a recent review [10] and summarized them in Supplementary Table S1. In the present review, we focus on the epigenetic mechanisms of neuroprotective actions of short peptides in AD that have not been previously reported.

The advantage of these compounds over other neuroprotectors is the lack of immunogenicity and a wide spectrum of action. It has been shown that ultrashort peptides are involved in the regulation of cell differentiation, apoptosis, and proliferation [10] and also have a neuroprotective effect in in vitro and in vivo AD models [13,14,15,16]. 

Previous studies have suggested an epigenetic mechanism of action of ultrashort peptides [17,18,19,20]. In this regard, the aim of the review is to analyze the possible epigenetic mechanisms of the neuroprotective action of short peptides in AD.

## 2. The Role of Short Peptides in the Pathophysiology of AD: Results and Prospects

**Figure 1:**
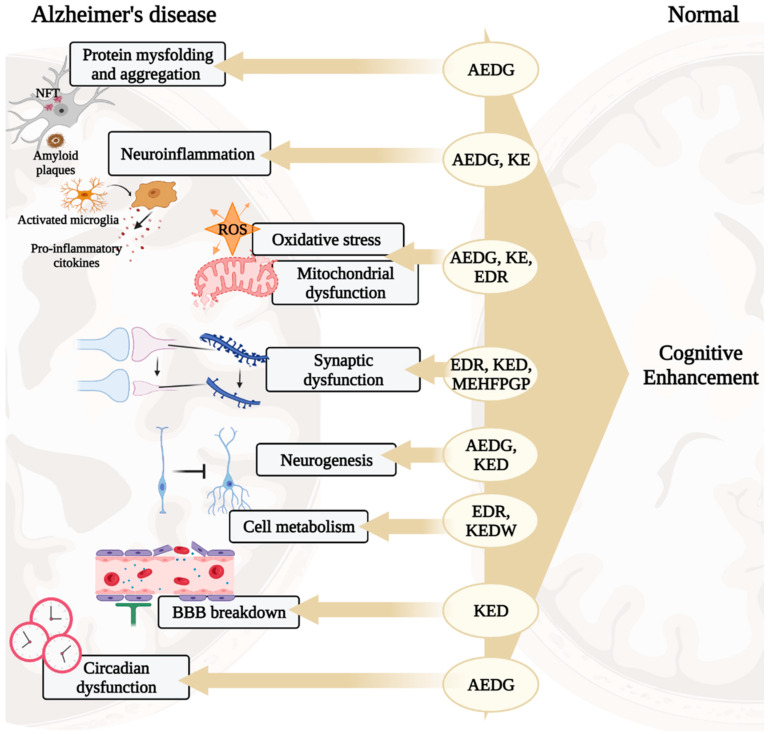
Potential neuroprotective roles of ultrashort peptides (KE, EDR, KED, AEDG, KEDW, and MEHFPGP) in AD. Note: BBB—blood–brain barrier, ROS—reactive oxygen species. (figure created with BioRender.com, accessed on 25 February 2022)

In the development of AD, there are such pathogenetic links as protein misfolding and aggregation, oxidative stress, mitochondrial dysfunction, neuroinflammation, impaired synaptic plasticity, etc. [2]. These processes are associated with the development of cognitive impairment manifesting in patients 10–15 years after the onset of the disease [21,22]. Short peptides are promising neuroprotectors, which is why their molecular genetic effects in the above processes will be considered below, schematically shown in Figure 1.

### 2.1. Protein Misfolding and Aggregation

The main histopathological features of AD are amyloid plaques and neurofibrillary tangles (NFTs), which are formed as a result of misfolding of the amyloid-β peptide (Aβ) and tau protein (τ). Aβ consists of 38–43 amino acid residues and is formed by sequential cleavage of a transmembrane amyloid precursor protein (APP) with a length of 695 amino acid residues, with the participation of β (BACE1)- and γ-secretases (contains proteins presenilins-1 (PSEN1) and presenilins-2 (PSEN2)). Autosomal dominant mutations in the APP, PSEN1, and PSEN2 protein genes that underlie the familial form of AD (FAD) increase the affinity of γ-secretase for APP, promoting the formation of Aβ [23,24].

Recent studies have shown that the life cycle of Aβ includes monomeric, oligomeric (AβO), and fibrillar forms (fAβ). Amyloid plaques are formed by fAβ, and their accumulation weakly correlates with cognitive impairment in AD [25]. Moreover, monomeric forms of Aβ predominate in the brain and cerebrospinal fluid, which, at critical nanomolar concentrations, due to the lack of a stable three-dimensional structure of Aβ, begin to aggregate with the formation of misfolded soluble oligomeric peptides (AβO) [26]. According to modern concepts, AβO plays a more significant role in AD pathogenesis than amyloid plaques, exerting a cytotoxic effect [27,28,29].

Furthermore, τ-protein stimulates the assembly of tubulin into microtubules in the brain [30]. The biological activity of the τ-protein is regulated by the state of its phosphorylation. In AD, abnormal hyperphosphorylation of the τ-protein leads to the formation of neurofibrillary tangles (NFTs), causing a cascade of pathological disorders in the functioning of the neuron [31]. AβO peptides also activate hyperphosphorylation of the τ-protein, thus indicating a pathogenetic relationship between the misfolding of Aβ and τ-protein [28].

Peptides are able to regulate the folding of the Aβ peptide and τ-protein in AD. It was found that the peptides Ac-Leu-Pro-Phe-Phe-Asp-NH2 (iAb5p), LPfFFD-PEG, D-(PGKLVYA), RI-OR2-TAT, cyclo(17, 21)-(Lys17, Asp21)Aβ (1-28), SEN304, SEN1576, α sheet peptides, NAP, D4-F, D3, PP-Leu, TFP5, and wtNBD inhibit AβO formation and Aβ aggregation into fibrils. NAP and TFP5 peptides attenuate τ-protein hyperphosphorylation [32]. Short amylin receptor antagonist peptides SQELHRLQTYPR (R5) and LGRLSQELHRLQTY (R14) have been shown to have a neuroprotective effect against Aβ toxicity, reduce neuroinflammation and restore neuroplasticity, and improve spatial memory in 5xFAD mice (AD model) [33].

Transthyretin (TTR) is one of the main Aβ-binding proteins that cleaves amyloid and thereby protects brain neurons from the neurotoxic effect of the Aβ peptide in AD [34,35]. Moreover, the pathological role of Aβ in the degenerative processes of the retinal pigment epithelium in the APPswe/PS1 transgenic mouse line (AD model) was revealed [36,37]. It has been shown that the AEDG peptide increases TTR synthesis in retinal pigment epithelium cells [38]. This indirectly indicates the potential ability of the AEDG peptide to prevent the accumulation of Aβ through the regulation of TTR synthesis.

The Aβ peptide was always the main target for the treatment of AD. However, this approach was not effective in clinical trials. Moreover, the Aβ peptide is present in many tissues outside the brain, including blood vessels, skin, subcutaneous tissues, intestines, and muscles [39], which probably causes the presence of side effects in targeted therapy for amyloidosis. It should be emphasized that a review of clinical trials over the past 5 years shows that more and more attention is being paid to “non-amyloid” targets, including possible treatments of inflammation, protection of synapses and neurons, regulation of vascular factors, mitochondrial function and oxidative stress, lipid and glucose metabolism, circadian rhythm, and neurogenesis, as well as epigenetic interventions [40].

### 2.2. Mitochondrial Dysfunction and Oxidative Stress

In addition to the classical bioenergetic function, mitochondria play an important role in calcium homeostasis in neurons [41], control of membrane excitability, neurotransmission, and plasticity [42]. Mitochondrial dysfunction is damage to the mitochondria that leads to changes in organoid permeability, disruption of the electron transport chain, and energy imbalance. At the same time, the accumulation of reactive oxygen species (ROS) is observed, causing the development of oxidative stress, including lipid peroxidation in the mitochondrial cell membrane. The release of cytochrome c, an element of the respiratory chain, triggers a cascade of caspases -3, -6, and -7, which contribute to the enzymatic cleavage of DNA and neuronal death [43].

The role of mitochondrial dysfunction in AD pathogenesis is presented by two hypotheses. Proponents of the theory of the mitochondrial cascade [44] indicate the primacy of mitochondrial disorders in AD pathogenesis, emphasizing the influence of ROS on APP processing [45] and dysregulation of mitochondrial processes in cytoplasmic hybrid (cybrid) cell lines containing mtDNA of AD patients. In such cybrids, an increase in the cytoplasmic content of cytochrome c; an increase in the activity of caspase-3 [46]; decreased bioenergetic function of neurons [47]; increased accumulation of oxidative stress markers; and activation of MAPK, Akt, and NF-kappa B [48] have been noted. In contrast, it is argued that mitochondrial bioenergetic dysfunction in AD is a consequence of a pathological Aβ-induced decrease in the activity of electron transport chain enzymes [49]. It was shown that Aβ interacts with cyclophilin D, an integral part of the mitochondrial permeability transition pore, the opening of which leads to cell death. This interaction potentiates mitochondrial dysfunction, synaptic dysfunction, and neuronal death [50]. Thus, mitochondrial dysfunction and oxidative stress can be considered as potential pathogenetic targets for therapeutic correction of the disease by using short peptides.

It was illustrated that intraperitoneal administration of the tetrapeptide SS-31 to Tg2576 mice (AD model) in the cerebral cortex reduces the level of the Aβ peptide and increases the level of mRNA and the synthesis of mitochondrial dynamic proteins (Mfn1/Mfn2), mitochondrial biogenesis (PGC1-α and Nrf2), and synaptic functions (synaptophysin and PSD95) [51]. As was analyzed, the SS-31 peptide regulates brain-derived neurotrophic factor (BDNF) signaling and improves learning and memory in the Morris water maze and the fear conditioning test that were impaired in mice under the condition of lipopolysaccharide-mediated oxidative stress (memory impairment model) [52].

Using DNA microarray technology, scientists have shown that the AEDG peptide changed the expression of 98 out of 15,247 heart and brain genes in mice, including genes encoding proteins responsible for mitochondrial functions: Hsp70, Hsp73, 16S, ND5, cytochrom b, and ND4. The same study found that the KE peptide reduced the expression of the *ATPase 6* gene [53]. These proteins are involved in key mitochondria-associated processes, such as energy metabolism and mitochondrial transport; for this reason, the short peptides AEDG and KE are probably promising neuroprotective compounds regulating the expression of mitochondrial functional state genes, a function that is important in the development of therapeutic strategy for AD treatment.

The EDR peptide has binding sites in the promoter region of the superoxide dismutase *SOD2* gene [13]. It was investigated that the levels of oxidative markers, including glutathione peroxidase and SOD, in the mitochondrial and synaptosome fractions in the human postmortem frontal cortex decreased and correlated with cognitive impairment in AD patients. These correlations highlight the impact of oxidative stress on synapse loss in AD [54]. In this regard, one of the mechanisms of the neuroprotective action of the EDR peptide may be its ability to regulate oxidative stress.

These data suggest that short peptides are potentially effective in correcting mitochondrial dysfunction and oxidative stress in AD.

### 2.3. Impaired Lipid and Glucose Metabolism

Lipids play a critical role in cell signaling and various physiological processes in the brain. There is evidence of a relationship between lipid metabolism in the brain and the development of AD [55]. Violation of glucose metabolism manifests itself in the form of type 2 diabetes mellitus. Epidemiological and pathophysiological studies have shown an association between AD and diabetes [56].

“Lipidosensitive” receptors activated by peroxisome proliferator (PPARα, PPARγ, and PPARδ) are involved in the regulation of lipid and glucose homeostasis [57]. The literature presents data on the role of these receptors in the development of neurodegenerative processes. It has been shown that pharmacological activation of PPARα receptors by their agonists gemfibrozil and 2-[4-chloro-6-(2,3-dimethylanilino)pyrimidin-2-yl]sulfanylacetic acid (Wy14643) induces autophagy in HM microglial and U251 human glioma cells expressing a mutant form of human APP (APP-p.M671L). The introduction of PPARα agonists reduces the accumulation of Aβ peptide, improves memory, and reduces anxiety in APP-PSEN1ΔE9 mice (AD model) [58]. In addition, it has been shown that PPAR-α downregulation is associated with a decrease in antioxidant and anti-inflammatory processes in the brain of patients with AD [59], thus highlighting the systemic nature of the neurodegenerative disease.

The use of the PPAR-γ agonist levistolide A in APP/PS1 Tg mice (AD model) led to a decrease in the number of amyloid plaques and a decrease in inflammation and the severity of behavioral disorders [60]. Badhwar et al. illustrated the role of PPAR-γ in the regulation of cerebrovascular pathology in AD. In particular, the PPAR-γ agonist pioglitazone has been shown to increase the synthesis of proteins responsible for the functioning of cerebral vessels [61]. In general, the literature repeatedly emphasizes the neuroprotective role of PPAR proteins in AD, as well as the prospects for the clinical use of their agonists. Thus, in a phase II clinical trial, the use of a PPAR-γ agonist improved cognitive function and memory in patients with mild-to-moderate AD [62].

Using the analysis of the promoter regions of the genes encoding proteins PPAR-α, PPAR-γ binding sites for the peptide EDR have been identified [63]. This indicates the potential ability of the tripeptide to have a neuroprotective effect by regulating the synthesis of PPAR-α and PPAR-γ proteins involved in AD pathogenesis.

It was shown that the KEDW peptide reduces the blood glucose level in rats under conditions of induced diabetes mellitus, and also regulates the expression of the *PDX1*, *NGN3*, and *PAX4* genes and the synthesis of the encoded proteins responsible for maintaining the functional activity of endocrine cells in the pancreas [64]. This indirectly indicates the potential ability of the KEDW peptide to regulate glucose metabolism in AD-associated diabetes mellitus.

Thus, since the energy balance of cells is closely related to synaptic activity and cognitive functions, and obesity and type 2 diabetes have been identified as risk factors for AD, the pharmacological targeting of PPARs may become one of the directions for the complex correction of neurodegenerative processes aggravated by metabolic disorders.

### 2.4. Neuroinflammation

Increasing evidence suggests that AD pathogenesis is not limited to the neuronal compartment and includes an immune component. Microglia are a special population of phagocytes migrating through the central nervous system (CNS) and distributed among the white and gray matter. In AD, microglia are activated via the binding of soluble Aβ oligomers and fibrils to CD36, CD14, integrin α6β1, CD47, and Toll-like receptors (TLR2, TLR4, TLR6, and TLR9). Activated microglia produce pro-inflammatory cytokines, including interleukin 1β (IL-1β) [65].

The pro-inflammatory cytokine IL-1β is activated in the early stages of AD development [66]. Elevated levels of IL-1β have been found in the prefrontal cortex and hippocampus in AD patients. It has been shown that IL-1β activates the expression of immune response, proliferation, and cytokine signaling genes inAPPswe/PS1dE9 mice (AD model) [67]. IL-1β stimulates APP secretion by glial cells and amyloidogenic APP processing by protein kinase C and γ-secretase. An increase in the concentration of Aβ42 under the action of IL-1β contributes to the activation of microglia and hyperproduction of IL-1β according to the principle of positive feedback [68]. The synthesis of IL-1β by microglia during the inflammatory process leads to the loss of dendritic spines, counteracting the stimulatory effect of BDNF [69].

There is a decrease in the expression of IL-1β mRNA in the cerebral cortex in 5xFAD mice [70]. In another study, enzyme immunoassay revealed an increase in the expression of IL-1β protein in the brain homogenate of 5xFAD mice [71].

IL-10 inhibits the synthesis of pro-inflammatory cytokines IL-1α, IL-1β, TNF-α, and IL-6 and the chemokine MCP-1 in the primary culture of microglia obtained from the cerebral cortex in the ND4 transgenic line of mice, which models neurodegenerative disorders during demyelination [68].

The AEDG peptide regulates the synthesis of IL-1β and IL-7 in thymic epithelial cells [72]. The KE peptide, being a fragment of the cytokines IL-lα, IL-2, IL-4, IL-5, IL-6, and INF-α, stimulates the synthesis of mRNA of the cytokine IL-2 in lymphoid cells [53] Consequently, it is suggested that the AEDG and KE peptides may have a neuroprotective effect through the regulation of neuroinflammation in AD.

Neuroinflammation in conjunction with oxidative stress contributes to dysfunction and death of oligodendrocytes involved in the formation of the myelin sheath of neurons. In addition, it was found that the Aβ peptide can disrupt the survival and maturation of oligodendrocyte progenitor cells and the formation of the myelin sheath of the neuron [73]. The formation of a pro-inflammatory phenotype of microglia is also associated with impaired neurogenesis, which is essential for maintaining the integrity of neural networks [74]. Loss of oligodendrocytes has been shown to correlate strongly with cognitive decline in AD [75].

Peptide 6, corresponding to the active region of ciliary neurotrophic factor, contributed to the recovery of cognitive functions in 3xTg mice (AD model) by enhancing neurogenesis in the dentate gyrus and neuroplasticity in the hippocampus and cerebral cortex [76]. It was also shown that peptide 6 reversed the loss of dendrites and synapses while increasing the activity of hippocampal trisynaptic circuits, leading to improved cognitive functions in C57Bl6 mice under conditions of head injury, which is a risk factor for AD [77].

The AEDG peptide has been shown to regulate the neurogenic differentiation of human periodontal ligament stem cells (hPDLSCs) by increasing mRNA expression and synthesis of Nestin, GAP43, β Tubulin III, and Doublecortin neurogenesis proteins [78]. The KED peptide has a similar effect [79]. Thus, the maintenance of neurogenesis and differentiation of oligodendrocytes by using peptides may underlie the strategy for stimulating the regenerative capacity of the brain as part of the therapeutic correction of AD.

### 2.5. Destruction of the Blood–Brain Barrier and Dysfunction of the Vascular System

The blood–brain barrier (BBB) is formed by a dense monolayer of brain endothelial cells that keeps neurotoxic plasma components, erythrocytes, leukocytes, and pathogens outside the CNS. Neuroimaging studies in individuals with mild cognitive impairment (MCI) and early AD have shown destruction of the BBB in the hippocampus and several regions of the gray and white matter of the brain [80]. Accumulation of Aβ peptide induces microvascular inflammation mediated by pro-inflammatory molecules activated in glia, endothelium, smooth muscle cells, and pericytes, and this is associated with cerebrovascular accident in AD [81]. The KED peptide had a normalizing effect on the state of capillary walls, increasing their resistance and permeability, and it also improved cerebral circulation [82]. Semax heptapeptide and PGP tripeptide stimulated the proliferation of neuroglia, vascular endothelium, and progenitor cells in the subventricular zone. At the same time, Semax reduced the manifestations of ischemic damage to the nervous tissue [83].

The vascular and neuronal components of the BBB interact with each other, secreting a number of trophic factors. Vascular function requires endothelial growth factor (VEGF). Normally, VEGF regulates microvascular density and controls vascular permeability, including that in the BBB. Neuron-specific overexpression of VEGF in APP/PS1 mice (AD model) partially eliminated the loss of cerebral vessels and restored cognitive functions. Administration of VEGF-releasing nanospheres to mice with AD contributed to neovascularization, decreased Aβ peptide aggregation, and reduced the severity of behavioral disorders [84]. It was shown that the KED peptide contributed to the restoration of VEGF expression in aortic endotheliocyte culture obtained from patients with atherosclerosis [85].

Thus, the neuroprotective effect of the KED peptide may be mediated by its influence on the expression of trophic factors, including VEGF. The obtained results indicate the expediency of further study of the KED peptide as a vasoactive component for the complex therapy of AD.

### 2.6. Synaptic Dysfunction and Neurotransmitter Balance

Synaptic dysfunction is one of the main factors in AD pathogenesis. Numerous studies have shown that the loss of synaptic contacts between neurons correlates with cognitive impairment in AD [86,87]. Previous studies have shown that the EDR peptide restored the number of dendritic spines in a culture of hippocampal neurons under conditions of amyloid synaptotoxicity (in vitro AD model) [14]. EDR and KED peptides prevented the elimination of mushroom spines in the 5xFAD line of mice [13].

Calmodulin (CALM) is the main Ca2+ binding protein in brain neurons which is involved in the regulation of the release of neurotransmitters from synapses; this process plays an important role in the synaptic plasticity and memory formation. In the promoter of the *CALM1* gene, binding sites for the EDR peptide have been identified which may determine the neuroprotective effect of the tripeptide in AD models [13]. In addition, it should be noted that, in neurons, the EDR peptide increases the activation of signaling mitogen-activated ERK1/2 kinase, the activity of which is of fundamental importance in the survival of neurons and synaptic plasticity [88].

It is noted that AD correlates with dysfunction of cholinergic and glutamatergic synapses in the early stages of the development [89]. The MEHFPGP peptide increased the survival of cholinergic neurons and stimulated choline acetyltransferase activity in dissociated cultures of neurons in the basal forebrain tissue [90].

Along with the loss of synapses, many neurotransmitters (including corticotropin releasing factor, somatostatin, GABA, and serotonin) become deficient as the disease progresses [89]. Mood disorders, including depression, affect up to 90% of AD patients [91]. Moreover, depressive behavior in mice is associated with a decrease in serotonergic tone in the brain [92]. It should be noted that the EDR peptide stimulates the synthesis of serotonin in the cerebral cortex cells [93].

Thus, the regulation of neurotransmitter synthesis, which underlies synaptic plasticity, and the normalization of the serotonergic status in the brain can become additional strategies for the neuroprotective action of short peptides in AD.

### 2.7. Circadian Rhythm Disruption

Circadian rhythms, defined as oscillations with a period of 24 h, are a fundamental component of mammalian physiology and serve to coordinate physiology with external signals, such as the light–dark cycle. The oscillations of the sleep–wake cycle are one of many systemic circadian processes in the body that are controlled by the suprachiasmatic nucleus of the hypothalamus (SCN). Circadian rhythms in the SCN and in most cells of the body, including neurons and astrocytes, are maintained by the core transcription machinery of the cell clock, consisting of the transcription factors BMAL1 and CLOCK, which heterodimerize and control the transcription of many genes, including their own negative feedback repressors, Period (Per) and Cryptochrome (Cry) [94].

Dysregulation of systemic circadian rhythms (e.g., sleep–wake cycle), melatonin activity, and secretion are common symptoms of dementia in AD [95]. It has been established that mutations in the *Per1* or *Per2* genes in mice are associated with impairments in spatial learning, conditional fear response, and long-term potentiation in the hippocampus [96]. *Bmal1* knockout (KO) mice exhibit astrocytosis, neuroinflammation, and degeneration of presynaptic endings [97], as well as impaired formation of long-term potentiation (LTP), contextual fear, and spatial memory [98]. It has been shown that the AEDG peptide modulates the expression of circadian genes, reducing the overexpression of the *Clock* and *Csnk1e* genes in leukocytes and increasing the hypoexpression of the *Cry2* gene in peripheral blood lymphocytes by two times in people with reduced melatonin-producing function of the pineal gland [99].

It has been established that melatonin plays an important role in neuroprotection during aging and mental disorders [100]. Melatonin improved memory in mice in an AD model by increasing *BDNF* and *CREB1* gene expression in the mouse prefrontal cortex [101]. It should be emphasized that the AEDG peptide restores the nocturnal peak of melatonin secretion in old monkeys (*Macaca mulatta*) to the norm of young animals [102].

Thus, the ability of the AEDG peptide to regulate the expression of circadian genes and melatonin synthesis may be an additional mechanism of the neuroprotective effect of this tetrapeptide in the complex correction of circadian rhythm disturbances in AD.

## 3. The Role of Short Peptides in Epigenetic Regulation of Gene Expression in AD

Epigenetic regulation of gene expression includes chromatin remodeling, DNA modification, ncRNA function, RNA methylation, and mitochondrial DNA methylation and hydroxymethylation [103]. Epigenetic processes interact closely to form a complex regulatory system that can dynamically tune gene expression. It should be pointed out that epigenetic regulation of neurodegenerative processes cannot only be directly related to the pathogenesis of the disease, but also mediate interactions between genetic and environmental risk factors for its development [104]. The latter can significantly complement the understanding of the pathogenesis of the sporadic form of AD that, in the epidemiological sense, prevails over hereditary AD and is more complex in terms of understanding its pathophysiology and potential therapy. In the context of the pathophysiology of AD, due to the fact that, at the present stage, there are no results linking additional epigenetic mechanisms with the pathogenesis of the disease, only the main mechanisms are considered here: chromatin remodeling, DNA methylation, and ncRNA [103].

**Figure 2:**
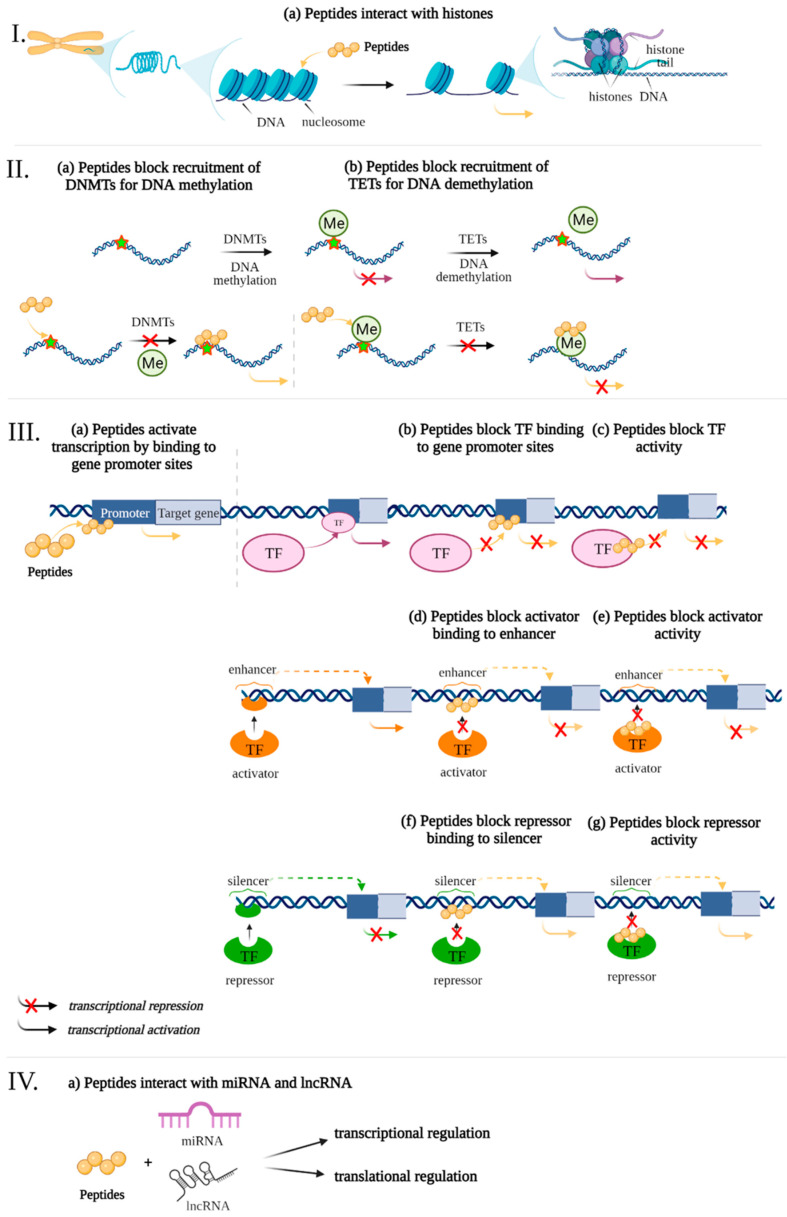
Potential epigenetic mechanisms of gene expression regulation by using ultrashort peptides. The levels of regulation: I—chromatin level, II—interference of peptides with DNA Methylation/Demethylation, III—interaction of short peptides with cis-regulatory elements of the genome and transcription factors, IV—RNA level. Note: DNMTs—DNA methyl- transferases, Me—methylation, TF—transcription factors, TETs—translocation enzymes. (figure created with BioRender.com, accessed on 25 February 2022)

Previously, data were presented that peptides regulate gene expression associated with the development of pathogenetic links of AD. The following is an analysis of potential epigenetic mechanisms of gene expression regulation by using short peptides (Figure 2).

### 3.1. Chromatin Level

The cellular genome is organized in the form of chromatin, a complex multilevel packaging of the DNA double-helix wound around histone proteins in combination with a large number of different regulatory factors [105]. The octamer, consisting of pairs of core histones H2A, H2B, H3, and H4 and a DNA strand of 146 base pairs (bp), makes up the nucleosome, which is the structural unit of chromatin. The winding of the DNA double helix around histones occurs due to the interaction of the negatively charged sugar-phosphate backbone of DNA with the positively charged amino acids of the histones. The linker and stabilizer of the DNA molecule in the nucleosome is histone H1. Compaction of DNA with histone proteins protects the molecule from inadvertent unwinding and copying. On the other hand, transcription and replication require a temporary separation of the two DNA strands, thus allowing polymerases to access the DNA template for transcription. Therefore, the degree of chromatin condensation affects its transcriptional activity. Thus, reversible histone modifications underlying the mechanisms of chromatin remodeling determine the availability of DNA for binding transcription factors and are a way to regulate gene expression [106].

The set of modifications of the N-terminal fragments of core histones that provide chromatin remodeling in the cell is called the histone code. The following modifications that make up the histone code are known: acetylation of lysine, methylation of lysine, arginine or histidine, phosphorylation of serine, threonine or tyrosine, ubiquitinylation, SUMOylation, ADP-ribosylation, crotonylation, hydroxylation, deamination, and isomerization of proline and lysine [103]. Most often, acetylation, methylation, phosphorylation, and ubiquitinylation are associated with activation of gene expression, while methylation, ubiquitinylation, SUMOylation, deamination, and proline isomerization may lead to gene repression [106]. A large number of combinations of histone modifications allow the histone code to flexibly adjust gene expression, which plays an important role in the regulation of brain neuroplasticity and is also associated with neurodegenerative processes [103].

Transcriptome analysis of post-mortem samples from the lateral temporal lobe of AD patients shows increased expression of genes associated with chromatin remodeling (genes encoding histone acetyltransferases CBP (CREBBP), p300 (EP300) and TRRAP (a subunit of the SAGA/ATAC complex), histone deacetylases SIRT1 and HDAC4, histone methyltransferases (CXXC31), and histone demethylases (JMJD6)). It was demonstrated that methylation sites (H4K20me2 and HK20me3) changed during aging and AD, while histone H3 acetylation increased only in AD (H3K27ac and H3K9ac) and was associated with such biological processes as transcription, nucleic acid metabolism, and Wnt signaling pathways that are involved in neurodegeneration, as well as synaptic transmission, neuronal death, immune response, and oxidative stress [107]. In another study, while evaluating genome-wide patterns of H3K27ac histone acetylation in samples of the entorhinal cortex of patients with AD, widespread acetyloma variations associated with the progression of tauopathy and accumulation of Aβ were identified [108]. These results indicate the important role of histone acetylation in the development of pathophysiological processes in AD.

HDAC inhibitors, including valproic acid (VPA), sodium 4-phenylbutyrate (4-PBA), vorinostat (SAHA), trichostatin A (TSA), and nicotinamide, have shown good results in AD mouse models [5]. Pharmacological histone deacetylation contributed to a decrease in the production of the Aβ [109], decrease in tau protein phosphorylation [110], increase in the stability of microtubules, restoration of dendritic spine density in the pyramidal neurons of the CA1 region of the hippocampus [110], and normalization of behavioral responses in mice in an AD model [109]. It allows us to conclude that the regulation of the pathogenetic links of AD at the level of chromatin remodeling may be one of the therapeutic strategies.

The ultrashort peptides AEDG and KE cause chromatin deheterochromatinization in the blood lymphocytes of elderly people [111,112]. Peptides AEDG, EDR, AEDL, KEDG, AEDR, and KEDW bind to FITC-labeled wheat histones H1, H2B, H3, and H4 in the peptide-binding motifs of the N-terminal regions [113]. It has been demonstrated by molecular modeling that the EDR and DS peptides can bind to histone H1.3. Such binding can affect the H1.3 histone conformation and lead to modification of the chromatin structure in the loci of the Fkbp1b gene. This gene encodes peptidyl-prolyl-cis-trans isomerase, which regulates the release of calcium ions from the sarcoplasmic and endoplasmic reticulum of neurons. Activation of transcription of the Fkbp1b gene through EDR- or DS-mediated activation of euchromatin can regulate calcium homeostasis in neurons, which is essential for leveling Ca2^+^-mediated neurodegenerative processes in AD [114]. It was found that the AEDG peptide can interact with histones H1/6 and H1/3 through the amino acid sequences His-Pro-Ser-Tyr-Met-Ala-His-Pro-Ala-Arg-Lys and Tyr-Arg-Lys-Thr-Gln that interact with DNA. Binding of the AEDG peptide and histones H1/3, H1/6 may determine the ability of the tetrapeptide to epigenetically regulate the expression of neuron differentiation genes and protein synthesis in human stem cells [78]. Thus, it is possible to trace the epigenetic mechanism of peptide regulation of the functional activity of neurons [10].

### 3.2. DNA Level

#### 3.2.1. Interference of Short Peptides with DNA Methylation/Demethylation

DNA methylation consists in attaching a methyl group to the fifth carbon atom of the nitrogenous base of cytosine in the DNA CpG dinucleotide to form 5-methylcytosine (5mC). Less common is the methylation of guanine and adenine with the formation of 7-methylguanine and 3-methyladenine, respectively. Overall, 70–80% of CpG sites are methylated, and the remaining unmethylated CpG sites are mostly found in dense clusters called CpG islands, which are often located in the promoter regions of genes [115]. The DNA methylation profile (methylome) is inherited (maintenance DNA methylation), while the formation of new sites is de novo DNA methylation; it occurs in the presence of DNA methyltransferase (DNMT) enzymes and S-adenosylmethionine (SAM) as a methyl group donor. Four types of DNA methyltransferases are known: DNMT1, DNMT2, DNMT3a, and DNMT3b. DNMT2 is a tRNA methyltransferase [116]. DNMT1 is responsible for maintaining pre-existing methylation patterns by methylating semi-methylated DNA after replication, while DNMT3 establishes new methylation patterns for previously unmethylated cytosines [117]. Most often, DNA methylation in the promoter region disrupts the binding of transcription factors to the gene, leads to transcriptional repression, and is mainly found in heterochromatin [103].

A number of studies have noted that DNA methylation is important in the regulation of Aβ peptide production. In particular, it has been shown that the promoter region of the *APP* gene is hypomethylated in patients with AD compared with a high level of methylation of this gene in the norm. Moreover, *APP* hypomethylation is associated with the overproduction of this protein and the Aβ peptide [118,119]. In another study, Aβ-mediated hypomethylation of the *heme oxygenase 1* (*HMOX1*) gene was noted to correlate with the development of cognitive impairment in AD [120]. The HMOX1 protein catabolizes heme to biliverdin, Fe^2+,^ carbon monoxide; its activity is increased in AD [121]. The Aβ peptide, along with inflammatory and oxidative stress, induces HMOX1 expression in astrocytes and neurons of the hippocampus and cerebral cortex in patients with AD [122,123].

Genomic and epigenomic analysis of 1.2 million CpG and CpH sites in enhancers of prefrontal cortex neurons in 101 people with no, mild, moderate, and severe AD showed that hypomethylation of enhancers in the *DSCAML1* gene (the protein encoded by this gene is a member of the Ig superfamily of cell-adhesion molecules and is involved in neuronal differentiation in AD is associated with the activation of BACE1 transcripts and an increase in the number of amyloid plaques, neurofibrillary tangles, and the development of cognitive impairment [124]. Another study showed that mRNA expression profiles of the IL-1β and IL-6 protein genes involved in the neuroinflammatory response in the brain of AD subjects are modulated by DNA methylation. This indicates a pathogenetic relationship between neuroinflammation and epigenetic modifications [125].

As it was noted earlier, cytosine methylation in CpG regions in DNA is one of the most common epigenetic DNA modifications and plays a key role in the pathophysiology of AD [5]. DNMT inhibitors are considered as regulators of neuronal [126] and hematopoietic [127] stem-cell differentiation. Thus, strategies aimed at the regulation of gene expression through modifications of DNA regions take place in the framework of a potential therapy for AD.

It was found that the ultrashort peptides AEDG and EDR bind to single- and double-stranded deoxyribonucleotides and regulate phage λ DNA hydrolysis, depending on the methylation status of CNG sites, which are targets for DNA cytosine methylation in eukaryotes [17]. The authors suggest that the peptides selectively bind to the CNG or CG promoter sites, making these sites inaccessible to DNA methyltransferases. As a result, the promoter remains unmethylated, which is a decisive element of gene activation [18]. Moreover, in cell cultures of the pancreas and bronchi, a modulating effect of the short peptides KEDW and AEDL on the expression of genes whose promoters had different methylation status during aging was revealed [19]. Probably, blocking the binding sites of DNA methyltransferases leads to a compensatory increase in the expression of their genes under the action of short peptides [20].

Although DNA methylation is stable inherited information, there are ten-eleven translocation (TET) enzymes that can remove methyl marks by oxidizing 5-methylcytosine to 5-hydroxymethylcytosine. It is assumed that DNA demethylation promotes transcriptional activation, since hydroxylation of 5-methylcytosine by TET1 promotes DNA demethylation in the adult brain [128]. Moreover, electroconvulsive induced expression of the Gadd45 gene involved in the removal of 5-methylcytosine DNA contributed to increased expression of the brain-derived neurotrophic factor and fibroblast growth factor genes in the hippocampus of adult mice [129]. Identified changes in the status of DNA demethylation in the hippocampus/parahippocampal gyrus of the brain of AD patients [130] suggest an important role of this epigenetic mechanism in the development of the disease. However, the small number of existing works requires further research.

On the other hand, it cannot be ruled out that ultrashort peptides, recognizing the methylated site of the promoter, make it inaccessible to the action of DNA demethylases, the pathological activity of which can enhance the expression of the target gene in AD [131]. Apparently, short peptides have the ability to modulate pathophysiological processes, depending on the level of pathology/state of the epigenetic apparatus, which is fundamentally important for the correction of various diseases, including AD. Such a principle seems more physiological and can minimize the number of side effects, a feat that is not currently achieved by FDA-approved drugs.

The ability of short peptides to regulate gene expression under various physiological and pathophysiological conditions has been the subject of a large number of works. A potential role of short peptides in the regulation of gene expression in AD can be assumed. There are reasons to believe that short peptides can be bound by regulatory DNA elements.

#### 3.2.2. Interaction of Short Peptides with Cis-Regulatory Elements of the Genome

Cis-regulatory elements (CREs) are regions of non-coding DNA that are involved in the regulation of transcription of neighboring genes. CREs include proximal (promoters) and distal (silencers and enhancers) regulatory elements [132]. It is important to note that background transcription activity is provided by a set of transcription factors common to all genes—general transcription factors (GTFs), whose activity is directed to gene promoters. Cell-type-specific and dynamic regulation of gene expression is carried out by using specific transcription factors (STFs), which bind 94% to distal regulatory elements and modulate the activity of promoters. Thus, STFs called repressors bind to silencers, while activators bind to enhancers [133].

A genome-wide study made it possible to identify active enrichment in epigenomic enhancers in the genes responsible for the functioning of the endolysosomal system in myeloid cells, and the authors proposed the consideration of its dysfunction in the context of AD pathogenesis [134]. It should be emphasized that the development of pathophysiological links in AD, including the production and clearance of the Aβ peptide in the brain, is associated with dysregulation of a large number of genes, the correction of which may underlie therapeutic strategies for AD.

Short peptides structurally and functionally remind zinc fingers, the DNA-binding domains of transcription factors. In particular, metal, iron, and zinc ions similarly affect the DNA binding parameters of a synthetic peptide containing the finger domain from the erythroid transcription factor GATA-1 [135], peptides (MBP-DF) [136], and the ultrashort peptide EDR [137].

In this regard, it is assumed that peptides can exhibit similar activity by binding to cis-regulatory elements of the genome (enhancers, silencers, and promoters). The results of studies are described, indicating that short peptides are able to bind to DNA at specific nucleotide sequences belonging to the promoter region of target genes [10]. It should be emphasized that this is a new approach to the analysis of functional domains (sites of ligands binding to DNA). Often, researchers solve the problem of identifying the functional features of domains in ligands (peptides, proteins) that bind to nucleic acids or proteins. Thus, we pay attention to a number of works that propose machine learning and bioinformatics tools for analyzing the binding of proteins to nucleic acids and peptides [138]; proteins binding to peptides [139]; amino acid residues binding to DNA, RNA, and proteins [140]; and prediction of small molecule binding sites in proteins [141]. On the other hand, we analyzed DNA segments with which short biologically active peptides bind. Using molecular dynamics methods, we were able to demonstrate that the EDR peptide interacts with the promoter regions of the genes involved in the pathogenesis of AD (*CASP3*, *TP53*, *SOD2*, *GPX1*, *PPARA*, *PPARG*, *NES*, *GAP43*, *SUMO1*, *APOE*, and *IGF1*) [13]. This review later describes this idea in more detail.

Previously, using molecular modeling methods, we identified the binding sites of the EW, KE, EDR, KED, AEDG, and KEDW peptides with double-stranded DNA (dsDNA) in the classical B-form. The Internal Coordinate Mechanics binding score (ICM-Score) was chosen as a parameter for assessing binding [142] (Table 1).

**Figure 3:**
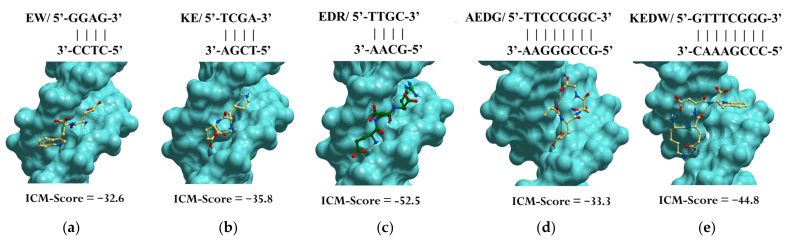
Site-specific binding of peptides EW (**a**), KE (**b**), EDR (**c**), AEDG (**d**), and KEDW (**e**) to double-stranded DNA in the classical B-form. Note: ICM-Score—the Internal Coordinate Mechanics binding score. Standard scheme of atom designation was following: C—yellow (green), N—blue, O—red.

When docking low-molecular-weight ligands, an ICM-Score ≤ −32 is acceptable for high-performance binding [142,143]. In this regard, a further analysis was carried out only for the peptides EW, KE, EDR, AEDG, and KEDW, which had the indicated binding score (Figure 3). It should be emphasized that di- and tripeptides predominantly bind in the minor groove of DNA, while tetrapeptides bind in the major groove. The KED peptide, which has a higher ICM-Score, regulates gene expression by a mechanism different from interaction with DNA in the classical B-form. For example, this can be mediated by interaction with histones and other functional molecules, as is discussed later in the review.

**Figure 4:**
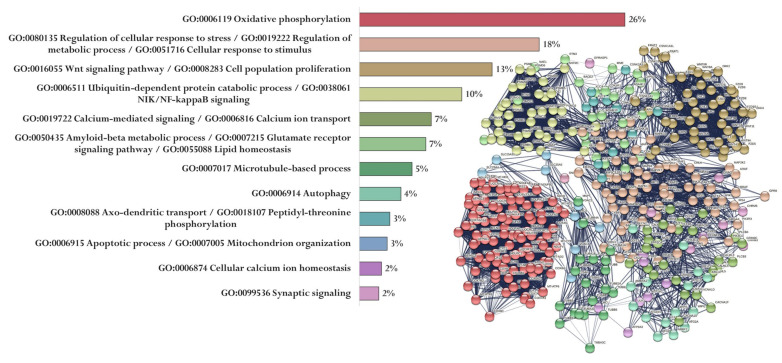
Functional relationship of proteins encoded by AD-associated genes according to the PathCards database [144]. Proportion of proteins in functional clusters (**left**). Protein–protein interaction networks (**right**).

Up to 8000 gene promoters containing the identified nucleotide sequences were found in The Eukaryotic Promoter Database. Each of the five peptides had its own set of promoters (Sets 1–5). At the same time, 369 genes (Set 6) associated with the development of AD were identified in the PathCards database. A cluster analysis (Markov Cluster Algorithm, inflation parameter—3, minimum required interaction score—0.4) of the functional relationship of proteins encoded by AD-associated genes was conducted by using the STRING database. The proteins were divided into 12 clusters (functional groups), which had the main GO terms associated with oxidative phosphorylation and the mitochondrial electron transport network, cell metabolism, cell proliferation, Wnt signaling pathway, functioning of the ubiquitin–proteasome system, synaptic neurotransmission, amyloid metabolism, cytoskeleton organization, autophagy, axonal transport, regulation of mitochondrial permeability, and Ca^2+^ signaling. It should be emphasized that only the key processes associated with the identified proteins are indicated here. The involvement of these proteins in different functional groups highlights the complexity of AD pathophysiology (Figure 4).

Next, a search was made for genes from Set 6 in Sets 1–5, thus making it possible to identify promoters involved in the development of AD from all the possible ones that are potentially binded to peptides. Thus, the share of promoters for peptides was 43% for EW, 8% for KE, 22% for EDR, 2% for AEDG, and 1% for KEDW.

**Figure 5:**
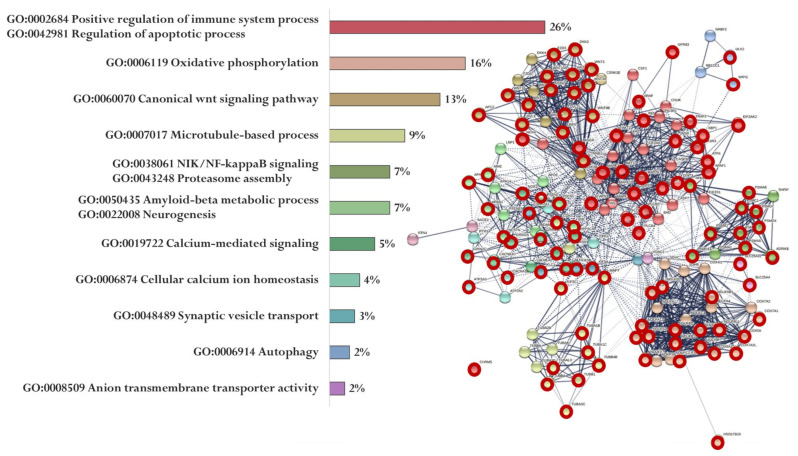
Functional relationship of proteins encoded by AD-associated genes—potential targets of the EW peptide (specific targets for EW peptide are marked with a red circle). Proportion of proteins in functional clusters (**left**). Protein–protein interaction networks (**right**): solid line shows the relationships between proteins within the same cluster, and dotted line shows the relationships between clusters.

**Figure 6:**
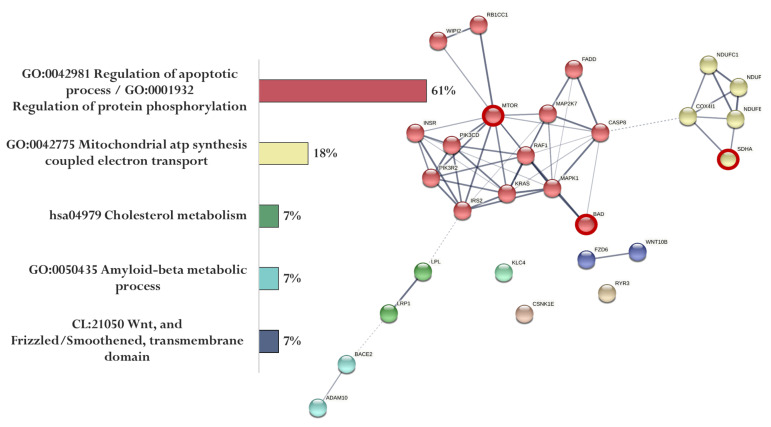
Functional relationship of proteins encoded by AD-associated genes—potential targets of the KE peptide (specific targets for KE peptide are marked with a red circle). Proportion of proteins in functional clusters (**left**). Protein–protein interaction networks (**right**): solid line shows the relationships between proteins within the same cluster, and dotted line shows the relationships between clusters.

**Figure 7:**
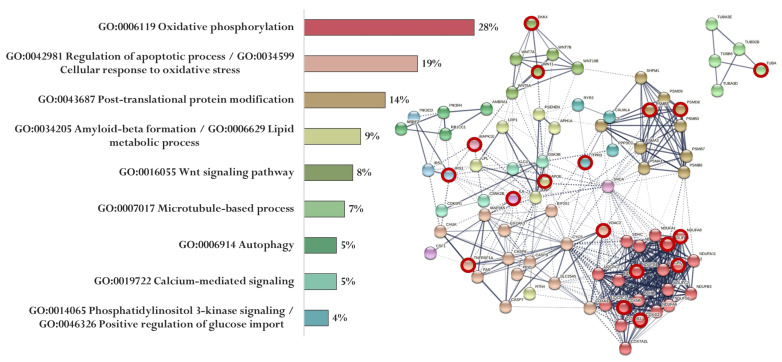
Functional relationship of proteins encoded by AD-associated genes—potential targets of the EDR peptide (specific targets for EDR peptide are marked with a red circle). Proportion of proteins in functional clusters (**left**). Protein–protein interaction networks (**right**): solid line shows the relationships between proteins within the same cluster, and dotted line shows the relationships between clusters.

Using the STRING database, we visualized relationships between proteins encoded by genes whose promoters bind the studied peptides. For each peptide, using cluster analysis (MCL clustering, inflation parameter—3, minimum required interaction score—0.4), a functional network of proteins was identified, and proteins whose genes are specific for a particular peptide (red circles) were also indicated (Figure 5, Figure 6 and Figure 7).

Figure 5 shows the functional relationship of proteins encoded by genes whose expression can potentially be regulated by the EW peptide (Figure 5). At the same time, genes specific for the EW peptide encode proteins involved in the regulation of the immune system process and apoptotic process (APAF1, ARAF, AGER, AKT2, NOX4, MAP2K1, AKT1, PIK3R1, INS, PLCB2, GPR83, XBP1, GPR83, ATF6, MAPK3, PLCB4, TRAF2, AGER, CAPN1, PIK3R1, AKT1, NOX4, EIF2AK2, PIK3R3, BID, CASP3, CHRM5, DDIT3, and PLCB1), oxidative phosphorylation (NDUFA13, NDUFA4L2, NDUFB10, NDUFB11, UQCRB, COX7A1, HSD17B10, NDUFB2, COX5A, COX7A2, NDUFA7, NDUFS7, NDUFA2, NDUFA9, COX6A2, NDUFAB1, NDUFS2, and NDUFB1), Wnt signaling pathway (WNT3, DVL3, AXIN2, DKK2, FZD3, WNT5B, WNT16, CTNNB1, LRP5, WNT2, APC2, DVL2, WNT3A, WNT10A, WNT9A, and WNT9B), microtubule-based process (TUBA1B, TUBB8, TUBAL3, CDK5, KIF5C, TUBB4B, MAPT, TUBA1C, TUBB1, and TUBA3C), NIK/NF-kappaB signaling Proteasome assembly (PSMC1, PSMA8, NFKB1, PSMA5, PSMD4, ADRM1, PSMA6, PSMD7, PSMA8, PSMB1, and PSMD14), Aβ metabolic process and neurogenesis (PSEN2, APBB1, PSEN1, IDE, PSEN2, APH1B, MME, and NCSTN), calcium-mediated signaling (GRIN2B, CACNA1C, NOS1, GRIN2A, CALM2, GRIN2C, PPP3CA, and CALM3), cellular calcium ion homeostasis (ATP2A2, CACNA1D, and ATP2A3), synaptic vesicle transport (KIF5, AKIF5B, and CSNK2A1), autophagy (WIPI1, TUBB8, and ULK2), and anion transmembrane transporter activity (SLC25A4 and SLC25A31).

The association of proteins encoded by genes whose expression can potentially be regulated by the KE peptide is shown in Figure 6. Genes specific for the KE peptide encode proteins involved in the regulation of apoptotic process and protein phosphorylation (MTOR and BAD) and mitochondrial ATP synthesis coupled with electron transport (SDHA) (Figure 6).

The association of proteins encoded by genes whose expression can potentially be regulated by the EDR peptide is shown in Figure 7. EDR peptide-specific genes encode proteins involved in oxidative phosphorylation (NDUFB8, NDUFA8, NDUFS4, NDUFA12, COX4I2, and COX5B), regulation of apoptotic process and cellular response to oxidative stress (TNFRSF1A, VDAC2), post-translational protein modification (PSMB5, PSMD8, PSMA3), Aβ formation and lipid metabolic process (APOE), Wnt signaling pathway (WNT1, DKK4), microtubule-based process (TUBA8), calcium-mediated signaling (ITPR3), phosphatidylinositol 3-kinase signaling, and positive regulation of glucose import (IRS1) (Figure 7).

For the AEDG peptide, the *PSMC6* and *ATG101* genes were identified, encoding proteins that are involved in the ATP-dependent degradation of ubiquitinylated proteins and autophagy, respectively. For the KEDW peptide, the *PSMC3* and *NDUFV3* genes encoding proteins that are involved in protein degradation and the functioning of the mitochondrial respiratory chain, respectively, were identified.

As a result of a cluster analysis for the peptides EW, KE, EDR, AEDG, and KEDW, genetic targets were identified that affect the key molecular mechanisms of neurodegenerative processes. The abundance of similar nonspecific targets indicates the general principles that can potentially underlie the regulation of gene expression by peptides in AD. At the same time, the identified specific targets for each peptide belonging to different pathophysiological parts of AD suggest that the study of the complex effect of short peptides can contribute to the creation of an effective therapeutic agent for the treatment of AD.

It should be emphasized that the charge of the listed peptides is negative, while the peptides have good binding parameters when analyzed in silico. Of course, this method of analysis requires further analysis in vitro; however, there is evidence of a negatively charged peptide C9, which specifically interacted with DNA [145]. This proves the possibility of charge redistribution due to the presence of a solvent and/or environmental elements in vivo and in silico in the presence of water molecules [146].

Thus, the potential ability of the peptides to interact with DNA regions located in the promoter regions of AD-associated genes was analyzed. The EW dipeptide and the EDR tripeptide have the highest potential activity in relation to the regulation of pathological processes in AD, but this needs to be confirmed in further studies.

As noted earlier, cis-regulatory elements also include enhancers and silencers [133]. It has been suggested that altered regulation of transcriptional enhancers plays a critical role in AD [147]. It cannot be ruled out that the sequences by which peptides bind to DNA may belong not only to promoters, but also to enhancers or silencers, thus increasing the range of potential mechanisms for the regulation of gene expression by short peptides. In this regard, in order to assess the potential binding of short peptides to the regulatory elements in the genome, it is necessary to carry out a functional analysis of the DNA sequences binding to short peptides in order to determine whether they belong not only to promoters, but also to other functional units.

#### 3.2.3. Interaction of Short Peptides with Transcription Factors

As was mentioned earlier, in the overwhelming majority of transcription factors, zinc fingers act as DNA-binding motifs. If, on the one hand, the zinc finger as part of the transcription factor interacts with DNA, and on the other hand, with the carrier itself, then, given the similarities between zinc fingers and short peptides, it cannot be ruled out that short peptides can also interact with transcription factors. There are data in the literature that indicate the possibility of peptides as “elements of protein chains” to bind to proteins [138]. A review, Inamoto et al. described examples of peptides that directly affect transcription factors [148].

The transcriptional adapter proteins CBP and p300 contain two copies of the TAZ zinc finger motif. A peptide derived from the p53 activation domain binds to a specific site on the surface of the TAZ2 domain [149] This indicates the ability of the peptides to regulate the activity of CBP and p300 proteins by binding to the TAZ zinc finger domain. Another study showed that small peptides with a protein dimerization motif but lacking a DNA-binding motif form non-functional heterodimers with the STF group, inhibiting their activity. This suggests that peptide-mediated control of transcription factor activity may be one of the mechanisms for regulating gene expression [150].

It was shown that ultrashort peptides regulate the hydrolysis of phage λ DNA by site-specific wheat endonucleases WEN1 and WEN2. This suggested that the peptides site-specifically bind to DNA regulatory elements and modulate the functions of DNA-binding proteins [18]. Moreover, it has been shown that the peptides modulate the expression of the *KNOX1* and *GRF* genes encoding DNA-binding proteins [151,152]. In this regard, it is possible that the peptide regulation of gene expression in AD may be mediated by the interaction of peptides with trans-regulatory elements of the genome. The meaning of the therapeutic strategy in AD may lie in the fact that peptides can bind not only to promoters/enhancers/silencers (cis-regulatory elements), but also to transactivating domains, thereby potentially activating silent genes, or repressor domains, and, as such, inhibiting the transcription of the target gene [145].

#### 3.2.4. Short Peptides and Zinc Fingers: Analogy or Epigenetic Hierarchy?

Transcription factors are the most abundant proteins containing zinc finger domains. However, given their great diversity, this group includes proteins with a wide range of molecular functions [153] aimed at regulating gene expression. The role of zinc finger proteins in the pathophysiology of neurodegenerative diseases is emphasized, and the pharmacogenetic aspects of their action underlie the creation of psychotropic drugs [154].

Zinc fingers have been repeatedly used as modular building blocks to create genetic constructs. It should be emphasized that the construction of DNA-binding domains based on zinc fingers is a promising technology for the creation of therapeutic compounds for various pathologies associated with changes in gene expression, including AD [155].

Peptides that can functionally replace the zinc finger domain were identified in a number of works using combinatorial libraries [156]. By adding functional groups to engineered DNA-binding domains, such as silencing domains, new transcription factors can be created to increase or decrease the expression of a target gene. It has been shown that a peptide containing three Zif268 zinc fingers can effectively repress transcription from RNA polymerase II promoters in vivo [157].

Due to the similar DNA binding ability of short peptides and zinc fingers, an additional strategy in the use of short peptides to regulate gene expression in AD may be the creation of transcription factor-type constructs with a short peptide as a DNA-binding motif. Probably, biocompatible short peptides can become a physiological alternative to genetically engineered products.

Cheng et al., in an attempt to identify peptides for functional replacement of zinc fingers in the transcription factor domain, found peptides that support transcriptional activation in vivo, but whose sequences do not resemble zinc fingers or any known DNA-binding domains in transcription factor [156]. This indicates that the peptides can also act as transcriptional transregulators.

There are no data in the literature on the mechanisms of regulation of zinc finger gene expression. However, when searching for DNA nucleotide sequences that bind short peptides, we found promoter regions of zinc finger proteins, PHD finger proteins, and ring finger proteins in the database of promoters. This makes it possible to not exclude the epigenetic hierarchy in the relationship between short peptides and zinc finger proteins. Possibly, the modulation of gene expression and zinc finger protein synthesis can mediate fine mechanisms of regulation of neurodegenerative processes with the help of short peptides.

### 3.3. RNA Level

According to modern concepts, only 2% of the transcribed genome is translated into proteins. Most of the non-translated DNA is involved in the regulation of gene expression in the form of ncRNA. There are about 20 types of ncRNA that are divided by size into small ncRNAs (sncRNAs), which consist of less than 200 nucleotides, and long ncRNAs (lncRNAs), which consist of more than 100,000 nucleotides. SncRNAs perform both structural (rRNA, tRNA, and snRNAs) and regulatory functions (miRNAs, siRNAs, snoRNAs, piRNAs, and spliRNAs), while lncRNAs are primarily regulatory [103]. Of the ncRNAs, miRNAs are described best [103]. Recently, miRNAs have attracted increasing attention in studies of AD pathogenesis [145]. Their genes are transcribed by RNA polymerase II, after which primary miRNA (pri-miRNA) is formed during post-transcriptional modifications. Pri-miRNA can contain up to six miRNA precursors (pre-miRNA), which are formed by cleavage of pri-miRNA by RNase III in the presence of nuclear proteins DGCR8 (DiGeorge Syndrome Critical Region 8) and Drosha. Sixteen percent of pre-miRNAs additionally undergo nuclear processing with the participation of adenosine deaminase acting on RNA (ADARs). With the help of the nucleocytoplasmic protein carrier Exportin-5, pre-miRNAs are exported from the nucleus to the cytoplasm, where they undergo final changes in the presence of Dicer endoribonuclease to obtain double-stranded mature miRNAs. Mature miRNAs regulate translation through RNA interference involving the RNA-induced silencing complex (RISC) or by binding to the 3’ untranslated region (3’-UTR) of the mRNA, which prevents translation initiation, and also regulate transcription by binding to gene promoters [158].

**Figure 8:**
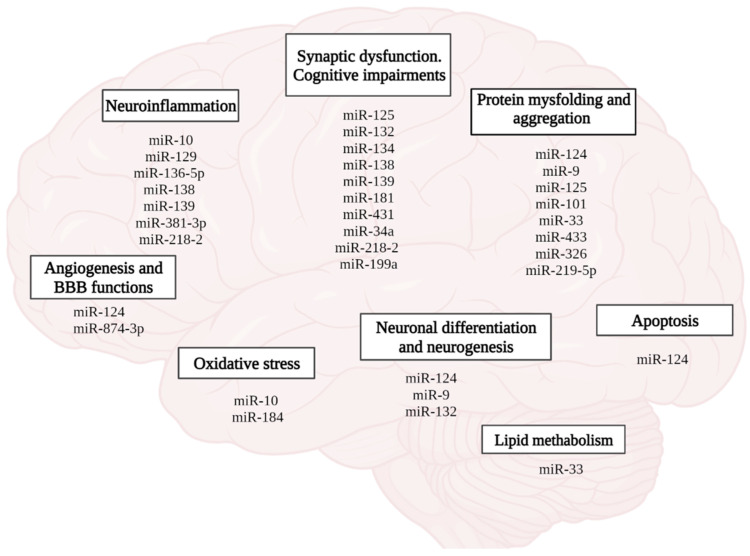
Role of miRNAs in the pathophysiology of AD. (figure created with BioRender.com, accessed on 25 February 2022)

Recently, the role of microRNAs in the formation of neuroplasticity, memory, and the development of AD has been emphasized (Figure 8). According to the PathCard database, there are about 50 types of microRNAs associated with the development of pathological processes in AD. In general, it should be noted that microRNAs mediate impaired neurogenesis, lipid metabolism, amyloid and tau protein, angiogenesis, development of neuroinflammation, and oxidative stress, which is accompanied by loss of synaptic contacts, impaired neuroplasticity and cognitive functions (Figure 8).

In the context of this review, attention should be paid to lncRNA, since lncRNAs are expressed in a cell-specific manner and may have protein-coding isoforms. LncRNAs regulate gene expression through several mechanisms at the level of transcription, translation, alternative splicing, and RNA processing [159].

LncRNA function is important in various biological processes, including AD [160]. It has been shown that the level of lncRNA 51A, transcribed into the intron of the low-density lipoprotein family receptor gene *SORL1*, is increased in the brain of patients with AD. Since SORL1 directs the cleavage of APP through the amyloidogenic pathway, elevated levels of 51A promote secretion of the Aβ peptide [161]. LncRNAs in the brain regulate the expression of neurotrophic factors, influencing the formation and function of synapses. BDNF, regulated by lncRNA BDNF-AS, maintains the survival and functional state of neurons, and also plays an important role in memory formation [162].

Interactions between nucleic acids and peptides were evolutionarily necessary to form the existing RNA/DNA/protein system. Modern research emphasizes the functional relationship between peptides and RNA [163]. It has been shown that protein cationic amino acids Arg, His, and Lys are chemically predisposed to the formation of longer linear oligomers than their non-protein counterpart’s ornithine, 2,4-diaminobutyric acid or 2,3-diaminopropionic acid, and can also generate depsipeptides that better stabilize RNA structures [163].

To date, peptides that specifically bind to RNA have been derived from proteins containing an arginine-rich RNA-binding motif. It is expected that other types of RNA-binding peptides (e.g., rich in aromatic residues) will be identified in other proteins through genetic and structural–functional analysis. Authors emphasize that, in order to improve binding depending on the target, peptides should also have a helical conformation stabilized by incorporating favorable charge interactions at the two ends of the helical dipole (alignment of peptide bonds in the helix generates a partial positive charge at the N-terminus and a partial negative charge at the C-terminus) [164]. Using peptide microarray technology, it has been shown that both hydrophobic and hydrophilic sides of short amphiphilic peptides are involved in interactions with RNA [165].

In the review, more attention was paid to negatively charged peptides. Despite the fact that RNA molecules are highly negatively charged, anions have been shown to bind to RNA [166], which does not exclude the possibility of binding of the considered peptides with RNA. Currently, approaches are being demonstrated to design peptides that bind to structured RNAs for microRNA biogenesis [167]. MicroRNAs, in turn, regulate the expression of the mRNA target gene by repressing translation. Two peptides, LKKLLKLLKKWLKLKG and LKKLLKLLKKLWKLKG, were found to bind to pre-miRNA-155, thereby downregulating Dicer-mediated miRNA-155 processing and upregulating the expression of miRNA-155 target genes in cells. Experimental results show that peptide inhibitors promote apoptotic cell death through a caspase-dependent pathway [168]. Undoubtedly, within the framework of AD, the therapeutic strategy should be directed, among other things, to the prevention of the apoptotic death of neurons. Probably, the search for the necessary miRNA as an effector element of interaction with peptides can contribute to the solution of this problem.

It should be noted that peptides can block the effector pathway of miRNA interaction with the target mRNA. It has been shown that a peptide from the GW protein required for the functioning of the Argonaute proteins (AGO) that mediate the interaction of miRNA with a target mRNA competes with endogenous GW proteins for binding to AGO and, thus, can be used as a potent inhibitor of the miRNA pathway. TNRC6 is one of the GW family proteins. It has recently been shown that a peptide derived from TNRC6B (amino acid position 599–683 in TNRC6B) competes with endogenous TNRC6 proteins for interaction with AGO and, thus, can effectively block miRNA-mediated target mRNA silencing [169].

It should not be ruled out that lncRNA can act as an intermediary between short peptides and the genome. LncRNA controls gene expression directly by binding to transcription factors of target genes and indirectly by complexing with other proteins to bind to target proteins and cause protein degradation, decrease in protein stability, or interfere with the binding of other proteins [170]. The review shows that lncRNA interacts with proteins [171]; therefore, it cannot be ruled out that the peptides can also actively bind lncRNA to regulate gene expression.

MiRNAs and lncRNAs have a potential coding role in terms of miRNAs-(miRNA-encoded peptides, miPEP) and lncRNAs-encoded peptides (lncPEP), starting a new phase of gene regulation [172,173]. Among miPEP and lncPEP, there are also short peptides that indicate the potential possibility of the neuroprotective peptides described in this review to realize their effect by analogy with these compounds. Moreover, miPEP and lncPEP are considered as a therapeutic basis obtained by genetic-engineering methods. In this regard, short neuroprotective peptides can become a promising replacement for genetically engineered constructs.

It should be emphasized that the principle of peptide regulation of gene expression can either consist in direct binding of short peptides to DNA or be mediated by interaction with effector molecules (transcription factors and DNA/RNA-binding proteins, including zinc finger proteins and ncRNA). In this regard, a proposed strategy for studying the epigenetic mechanisms of peptide regulation of gene expression can be a functional analysis of peptide–DNA binding regions, as well as a comparison of peptide structures with functional domains of regulatory molecules (transcription factors, DNA/RNA-binding proteins, and ncRNA) [174], directly or indirectly related to the regulation of gene expression in AD and other neuropathology.

## 4. Concluding Remarks and Future Directions

According to the latest data, worldwide studies of AD emphasize the need for a multifunctional and systemic approach to studying the pathogenesis of this disease. Moreover, transcriptomic studies suggest that AD pathogenesis consists of a wide range of intersecting molecular pathways [175]. This review considered such pathophysiological links in the development of AD as misfolding and aggregation of τ-protein and Aβ peptide, mitochondrial dysfunction, oxidative stress, impaired neurogenesis, glucose and lipid metabolism, neuroinflammation, and destruction of the BBB. These processes contribute to the disruption of the functioning of neural networks with the subsequent development of cognitive dysfunction and memory loss, the main symptoms of AD, recorded 10–15 years after the onset of primary molecular disorders.

Epigenetic mechanisms of gene-expression regulation play a key role in the functioning of neuronal plasticity in normal conditions and in the development of neurodegenerative pathologies, including AD. In earlier studies on various model systems, ultrashort peptides were characterized as biologically active compounds with the broad spectrum of activity, without reported side effects and an epigenetic mechanism of gene-expression regulation. In addition, in studies on models of AD in vitro and in vivo, short peptides showed a pronounced neuroprotective effect [13,14]. In this regard, in this review, we analyzed the epigenetic mechanisms of peptide regulation associated with AD pathogenesis.

**Figure 9:**
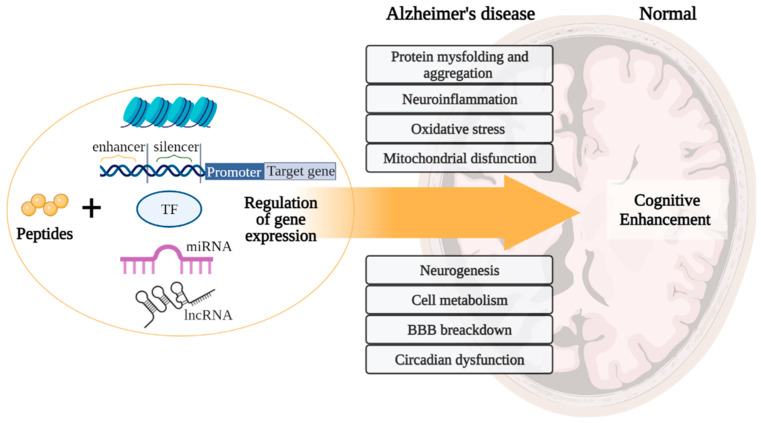
Concept of peptide regulation of neurodegenerative processes in Alzheimer’s disease. Note: BBB—blood–brain barrier. (figure created with BioRender.com, accessed on 25 February 2022)

Based on the general principles of peptide regulation, there is reason to believe that short peptides can realize their effects at the epigenetic level through interaction with histone proteins, cis- and transregulatory DNA elements, and ncRNA. Moreover, due to the similarity of short peptides with zinc finger domains in transcription factors, it is possible that short peptides can become an alternative to genetic constructs in artificial transcription factors for the purpose of site-directed regulation of gene expression in various pathologies, including AD. In general, a further comparative analysis of the structure of short peptides with functional domains of regulatory molecules (transcription factors, DNA/RNA-binding proteins, ncRNA) will allow us to reveal the key effector links of peptide regulation of gene expression. Figure 9 proposes a concept that covers all the considered potential targets of action of short peptides, as well as their relationship with modern molecular cellular pathophysiological parts of AD. We suggest that binding short peptides with nucleosome and/or promoters/enhancers/silencers and/or transcription factors and/or miRNA and/or lncRNA underlies the mechanisms of regulation of gene expression involved in such processes as protein aggregation, neuroinflammation, oxidative stress, mitochondrial dysfunction, neurogenesis, cell metabolism, BBB breakdown, and circadian dysfunction. That is, short peptides probably modulate neurodegenerative processes at the epigenetic level, thus contributing to the improvement of cognitive functions in the AD and constituting the cornerstone of neuroprotection. This concept is the basis for further research in order to create an effective polyfunctional drug based on short peptides for AD therapy.

## Figures and Tables

**Table 1:** Assessment of binding of short peptides to dsDNA in the classical B-form.

Peptide	DsDNA Sequence	ICM-Score
EW	GGAG	−32.6
KE	TCGA	−35.8
EDR	TTGC	−52.5
KED	TTAGGG	−31.7
AEDG	TTCCCGGC	−33.3
KEDW	GTTTCGGG	−44.8

## Data Availability

PathCards database, https://pathcards.genecards.org/Pathway/2022 (accessed on 20 September 2021); STRING database https://string-db.org (accessed on 20 September 2021).

## Supplementary Materials

The following are available online at https://www.mdpi.com/article/10.3390/ijms23084259/s1. References [10,13,14,32,33,38,53,63,64,72,78,79,85,88,90,93,99,114] are cited in the supplementary materials.
