# Supplementary material for: Neuroepigenetic Mechanisms of Action of Ultrashort Peptides in Alzheimer’s Disease
Source: Int J Mol Sci. 2022 Apr 12;23(8):4259. doi: 10.3390/ijms23084259 (PMC9032300; doi:10.3390/ijms23084259)
Supplement: Supplementary file 1 [file ijms-23-04259-s001.zip › ijms-1649099-supplementary.pdf]

Table S1. Role of short peptides

N	Structure of Peptides (Name)	Biological role of Peptides	References
1	AEDG (Epitalon)	retina-protective effect, antioxidant effect, stress-protective effect, geroprotection, activation of skin fibroblasts function, differentiation of plant cells, neurogenesis, Aβ aggregation, mitochondrial functions, neuroinflammation (synthesis of IL-1β and IL-7), circadian rithm	[10, 38, 53, 72, 78, 99]
2	DS	calcium homeostasis	[114]
3	EDR (Pinealon)	calcium homeostasis, synthesis of serotonin, neuroprotection, neuronal differentiation, antioxidant effect	[10, 13, 14, 63, 88, 93, 114]
4	EW (Thymogen)	regulation of immune system function, antioxidant effect, stress-protective effect, geroprotection	[10]
5	KE (Vilon)	regulation of immune system function, antioxidant effect, stress-protective effect, geroprotection, neuronal differentiation, differentiation of plant cells	[10]
6	KED (Vesugen)	regulation of cardiovascular system function, neuroprotection, neuronal differentiation, activation of skin fibroblasts' function, geroprotection	[10, 13, 79, 85]
7	KEDW (Pancragen)	regulation of glucose metabolism	[64].
8	MEHFPGP (Semax)	neuroprotection	[10, 90]
9	Ac-LCFFD-NH2 (iAb5p)	Aβ aggregation	[32, 33]
10	LPfFFD-PEG,		
11	D-(PGKLVYA)		
12	Ac-rGffvlkGrrrrqrrkkrGy-NH(2) (RI-OR2-TAT)		
13	cyclo(17, 21)-(Lys17, Asp21)Aβ (1-28)		
14	d-[(chG)-(Y)-(chG)-(chG)-(mL]-NH(2)		
15	(SEN1576)		
16	(D4-F)		
17	(D3)		
18	(PP-Leu)		
19	(wtNBD)		
20	SQELHRLQTYPR (R5)		

21	LGRLSQELHRLQTY (R14)		
22	NAPVSIPQ (NAP)		
23	(TFP5)	Aβ aggregation, τ-protein hyperphosphorylation	[32]
